# Effect of diet composition on glandular gastric disease in horses

**DOI:** 10.1111/jvim.16747

**Published:** 2023-06-01

**Authors:** Samy Julliand, Marjorie Buttet, Tanguy Hermange, Patrick Hillon, Véronique Julliand

**Affiliations:** ^1^ UMR PAM A 02.102 Dijon France; ^2^ Lab To Field Dijon France; ^3^ Centre Hospitalier Vétérinaire Equin de Livet Livarot‐Pays‐d'Auge France; ^4^ University of Bourgogne‐Franche Comté Dijon France; ^5^ INSERM U1231, Lipids, Nutrition, Cancer Dijon France; ^6^ Department of Hepatogastroenterology University Hospital Dijon France; ^7^ Agrosup Dijon Dijon France

**Keywords:** buffering capacity, French trotters, gastric ulcer, pelleted alfalfa, simple sugars

## Abstract

**Background:**

Nutritional factors are suggested to influence the incidence and severity of glandular gastric disease (GGD) in horses.

**Objectives:**

To retrospectively assess whether dietary fermentable carbohydrates increase the severity of GGD and to prospectively evaluate whether the partial substitution of concentrates by dehydrated alfalfa would decrease GGD severity scores.

**Animals:**

In total, 82 trotters from 4 training centers exercised ≥5 days/week.

**Methods:**

Multicenter retrospective observational study, and prospective 2‐arm randomized trial. Glandular mucosae were observed by gastroscopy and scored (0‐4 severity scale) at day 0 (D0). Biochemical composition of the diet fed was compared between ulcerated and nonulcerated groups. After D0, horses either received the same diet (control, n = 41) or pelleted dehydrated alfalfa substituting 50% concentrates (alfalfa, n = 41). Glandular scores were recorded in both groups after 21 (D21) and 42 days (D42). The first end point was a successful outcome, defined as a horse with a glandular score of 2 to 4 on D0, decreasing to a score of 0 to 1 on days 21 or 42.

**Results:**

Horses scored 0 to 1 at D0 ingested more (*P* = .01) soluble sugars from concentrates than those scored 2 to 4 before D0 (77.5 g/kg BW; 95% confidence interval [CI]: 71.1‐84.0, vs 59.1 g/kg BW; 95% CI: 48.0‐70.3), whereas starch intake did not differ between groups (*P* = .24). Among horses scored 2 to 4 at D0, fewer were scored 2 to 4 in the alfalfa group (1 out of 6) compared with the control group (6 out of 6) at D42 (*P* = .02). Clinical success was 47.7 times more likely in horses fed alfalfa compared with horses in the control group (95% CI: 1.6‐1422.8).

**Conclusion and Clinical Importance:**

Relationships were found between diet composition and integrity of the glandular mucosa. Feeding pelleted dehydrated alfalfa could help to reduce the incidence and severity of GGD.

**Abbreviations**
ADF: acid detergent fiber
BW: body weight
DM: dry matter
GGD: gastric glandular disease
IV: intravenous
NDF: neutral detergent fiber
SGD: squamous gastric disease

## 1 INTRODUCTION

In racehorses, gastric ulcers affecting the glandular region, defined as glandular gastric disease (GGD), are associated with performance that is below expectation.^1^ Using a 0 to 4 grading system, the prevalence of GGD graded ≥2 can exceed 50% in racehorses^2^ and sport horses.^3^ Some nutritional factors and practices, such as providing electrolytes^4^ and feeding high amount of starch^5^ are suggested to increase the risk of GGD. Horses fed a high‐starch diet for 72 days have more glandular ulcers than those fed a high‐fiber diet. Furthermore, after 30 days of grain fractionating over 20 meals per day, the proportion of horses with GGD is lower compared with the group fed twice daily, and no difference is detected after 60 days.^6^ Although a link with dietary practices is not consistently observed,^1^ these results suggest that dietary carbohydrates might influence development of GGD. Thus, our first objective was to evaluate whether higher fermentable carbohydrate intake was related to higher GGD scores in a cohort of horses at training.

Exposure to short‐chain fatty acids (SCFAs), originating mainly from starch and sugar fermentation in the stomach, can be detrimental to the glandular mucosa in acidic conditions. In monogastric animals, acetic acid is used as a model for inducing glandular ulcers,^7^ and in horses, butyric acid impairs mucosal integrity in vitro under low pH.^8^ Therefore, regulating gastric acidity seems important and acid‐suppressing treatment is indicated in the management of GGD in horses, with omeprazole being the drug of choice.^9^ However, GGD is less responsive to proton‐pump inhibitor monotherapy than is squamous gastric disease (SGD) in horses.^10^ Manipulating gastric pH can also be achieved through dietary solutions, such as using high‐buffering feedstuffs. Among plants commonly fed to horses, alfalfa is one of the most effective antacids.^11^ In a gastric environment, alfalfa maintains a higher pH in vivo^12^ and in vitro.^13^ Feeding alfalfa hay to yearlings for 28 days is associated with a lower ulcer severity score than feeding costal Bermudagrass hay.^14^ Feeding alfalfa forage‐based feeds for 42 days reduces ulcer severity scores in both the glandular and nonglandular areas in horses not at work.^15^ However, these findings remain controversial as more severe mucosal lesions at the pylorus are found in weanling horses fed alfalfa chaff for 16 days after weaning.^16^ There are no differences in other gastric regions when feeding alfalfa chaff and no detrimental effect at the pylorus when feeding alfalfa pellets mainly made of small particles.^16^ Therefore, we hypothesized that replacing part of the concentrates in the ration with alfalfa pellets might improve GGD scores. Thus, our second objective was to determine whether substituting part of the concentrates with alfalfa pellets would decrease GGD scores in a group of horses at training.

## 2 MATERIALS AND METHODS

Horses in training for trotting racing were recruited in 4 training centers. Approval for examination was obtained from the trainers before the start of the study. The referring veterinary clinic of the 4 training centers was the Centre Hospitalier Vétérinaire Equin de Livet. The study was conducted between January and February 2020, in accordance with applicable regulations at that time. The protocol was approved by the local ethics committee for animal experimentation of the University of Burgundy (*Comité d'éthique de l'expérimentation animale grand campus Dijon*) under the number 2019589602. All procedures were done with respect to the animal welfare by an equine veterinary practitioner specialist in internal medicine.

### 2.1 Study design

The study cohort comprised 82 French Trotters (9 geldings, 34 stallions, 39 mares), aged 2 to 3 years, and weighing 336 to 592 kg (436 ± 42 kg body weight [BW]). Horses were recruited in 4 training centers that had comparable training and feeding management. They were exercised 5 to 7 days per week, including 1 to 3 days of fast exercise on a track per week.

In each training center, 2 homogeneous groups of horses were formed based on their basal ulcer scores at day 0 (D0), age, and sex. The groups were then randomly assigned using a number generator to the “control group” or the “alfalfa group.” Apart from the diet, the randomized trial was conducted similarly for the 2 groups for 42 days. Before the beginning of the trial, it was decided that horses that stopped training, had signs of colic, were treated with nonsteroidal anti‐inflammatory drugs, or consume less than half of the amount of alfalfa distributed would be withdrawn from the trial.

### 2.2 Animal management and diet

All horses were housed in individual boxes on straw bedding, with free access to water and a mineral salt block. Trainers were requested to feed their horses the same diet for 4 weeks before D0. This diet consisted of the control diet, which was maintained afterward in the control group. The trainers maintained the same workload for all horses during the trial. In the 4 training centers, horses had ad libitum access to hay, and the concentrates were distributed in 3 daily meals (morning, noon, and afternoon). A person was designated in each training center as the referent for the preparation and distribution of meals. Concentrates were fed using the same scoop throughout the trial, the content of which had been measured. When refusals of concentrates were observed before the morning meal, the quantity that had not been ingested was recorded. Hay consumption by horses was estimated in each center from the weight of hay bales and the number of days recorded between the distribution of 2 bales. In 3 training centers, horses had limited daily access to outdoor paddocks.

From day 1 (D1) to day 42 (D42), horses in the control group received the same ration as before the beginning of the trial. Half of the initial concentrates in the alfalfa group were gradually substituted (v/v) by dehydrated alfalfa pellets over 3 days. Total nutrient intake was calculated from biochemical analyzes of all feedstuffs (Dairy One, Ithaca, NY; Table 1). The day before the dietary change (D0), and then at D21 and D42, horses were weighed, and their body condition score (BCS) was assessed by a veterinary practitioner on a scale of 1 (emaciated) to 9 (obese).^17^

**TABLE 1:** Estimated daily composition of the ration and nutrient intakes for the intensively exercised trotters that completed the trial in the 4 training centers (means ± SD).

	Group control		Group alfalfa	
Composition of the ration				
Hay (kg DM)	7.9 ± 1.6		7.9 ± 1.6	
Concentrates (kg DM)	5.7 ± 1.6		3.0 ± 0.8	
Pelleted alfalfa (kg DM)	0		2.8 ± 0.6	

Energy and nutrients intakes	From concentrates	From hay + concentrates	From concentrates	From hay + concentrates
Digestible energy (MJ)	81 ± 23	149 ± 23	67 ± 17	134 ± 20
Crude proteins (g)	773 ± 229	1426 ± 300	825 ± 223	1481 ± 295
Calcium (g)	62 ± 23	94 ± 20	86 ± 28	117 ± 23
Phosphorus (g)	34 ± 10	54 ± 10	25 ± 6	46 ± 8
Starch (g)	1894 ± 644	1925 ± 627	1030 ± 319	1060 ± 312
Simple sugars (g)	311 ± 104	895 ± 110	285 ± 80	860 ± 118
NDF (g)	1586 ± 338	7170 ± 764	2116 ± 524	7606 ± 810
ADF (g)	884 ± 294	4364 ± 416	1473 ± 429	4890 ± 432

Abbreviations: ADF, acid detergent fibers; DM, dry matter; NDF, neutral detergent fibers.

### 2.3 Gastroscopy and GGD scoring

The gastroscopic examination was performed for all horses at D0, D21, and D42. Muzzles were fitted to the individuals the evening before the examination to improve gastric visualization, but there was no water access restriction. For the examination, horses were sedated with a combination of detomidine (Sedomidine; Laboratoire Audevard, Clichy, France; 0.01 mg/kg IV) and butorphanol (Torbugesic; Zoetis, Malakoff, France; 0.01 mg/kg IV), and a 3.2‐m‐long video endoscope (Optomed, Les Ulis, France) was introduced into the stomach. After insufflation and rinsing adherent food material from the mucosa, the glandular mucosa was observed, including the antrum and the pylorus. A single experienced internal medicine board‐certified equine veterinary practitioner blinded to the treatment group scored the entire glandular mucosa during each gastroscopy. A 0 to 4 scoring system was used (Table 2), as adapted from Sykes and Jokisalo.^18^ In addition, the endoscopic features of 3 mucosal regions (glandular fundus, antrum, and pylorus) were qualitatively recorded using different criteria, namely congestion (red, swollen, and thickened appearance of gastric folds), hemorrhage, fibrinous area, and mucosal relief (not flat appearance).

**TABLE 2:** The grading system for the scoring of the glandular mucosa (adapted from Reference 18).

Glandular mucosa score	Description
0	Intact normal mucosa, without evidence of hyperemia
1	Intact mucosa, with areas of hyperemia
2	Small, single or multifocal superficial lesions
3	Large single deep or multifocal superficial lesions
4	Extensive lesions with areas of evident deep ulceration

### 2.4 Data analysis

The effect of diet on changes in BW and BCS was assessed using mixed‐effect models with a normal distribution of errors in a MIXED procedure (v9.3, SAS Institute Inc., Cary, NC). Day, Group, and the interaction Day × Group were included as fixed effects, and Day was included as a repeated measure per horse.

Gastric glandular scores were categorized into physiological (0‐1; “nonulcerated”) or clinically relevant (2‐4; “ulcerated”) groups. First, we assessed the relationship between diet fed before the beginning of the trial and GGD at D0. For that purpose, we compared dietary intake at D0 for the nonulcerated and ulcerated groups using the LSMEANS option in a MIXED procedure with a Tukey adjustment (SAS v9.3). Then, the second outcome was to estimate the success in preventing GGD incidence at D21 or D42, defined as remaining in the nonulcerated group for the horses that were not ulcerated at D0. Finally, the clinical success in the ulcerated group at D0 was evaluated. This curative success was defined as transitioning from the ulcerated group at D0 to the nonulcerated group at D21 or D42. The difference in the proportions of success and failure according to the diet was compared at D21 and D42 with a Fisher exact test. The odds ratio and 95% confidence intervals (CI) were calculated (SAS v9.3). The level of significance was set at *P* < .05.

## 3 RESULTS

### 3.1 Horses

**FIGURE 1:**
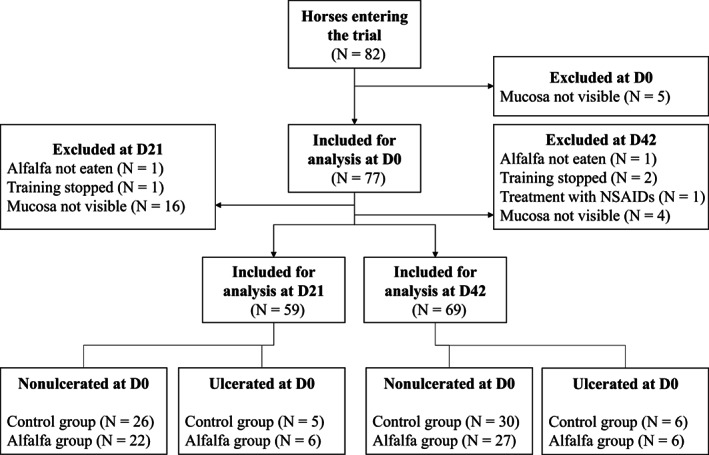
Flowchart of the trial selection process showing the number of horses at each step.

Body weight and BCS of horses were similar between groups and did not vary during the 42 days. Two horses were excluded from the D21 analysis based on the criteria defined before the beginning of the trial, and 2 additional horses were excluded from the D42 analysis (Figure 1). Glandular mucosa could not be observed clearly and scored because of an abundant gastric lake in 5 horses at D0, 16 horses at D21 (8 in the control group, 8 in the alfalfa group) and 4 horses at D42 (2 in each group).

### 3.2 Prevalence of GGD and mucosa endoscopic features

At D0, glandular scores ≥2 were observed in 13 of the 77 horses whose glandular mucosa was observed (prevalence 17%; 95% CI: 9%‐25%). At D21, the number of individuals with GGD score ≥2 was 3 of 31 horses (prevalence 10%; 95% CI: 0%‐20%) in control group. No individual (0/28) was observed with GGD score ≥2 in the alfalfa group. At D42, the number of individuals with GGD score ≥2 was 12 of 36 horses (prevalence 33%; 95% CI: 18%‐49%) in the control group and 4 of 33 (prevalence 12%; 95% CI: 1%‐23%) in the alfalfa group. No horse developed GGD graded 4 during the trial.

**FIGURE 2:**
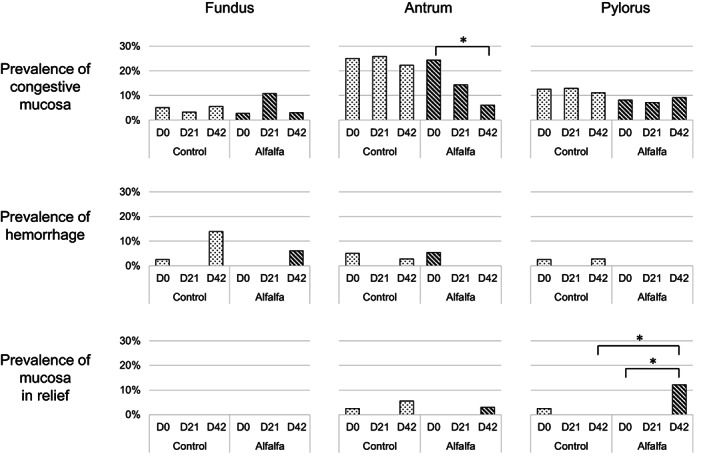
The proportion of congestive mucosa, hemorrhage, and mucosa in relief (not flat appearance) in the fundic, antral, and pyloric regions observed during gastroscopies at D0, D21, and D42 in intensively exercised trotters fed either the control diet (hay ad libitum +5.7 ± 1.6 kg DM concentrates) or the alfalfa diet (hay ad libitum +3.0 ± 0.8 kg DM concentrates +2.8 ± 0.6 kg DM alfalfa pellets) after D0. Horizontal lines indicate test cut‐off. * *P* < .05.

Congestive mucosa was the most recorded abnormal observation during the trial compared with hemorrhage, mucosa in relief, and the presence of a fibrinous area (Figure 2). It was predominantly located in the antral region (prevalence 25% at D0; 95% CI: 15%‐34%) and the pylorus (prevalence 12% at D0; 95% CI: 5%‐19%). Concomitant congestion of these 2 regions was observed in 2 horses at D0, 1 horse at D21, and 2 horses at D42. The proportion of congestive mucosa in the antral region decreased between D0 (prevalence 24%; 95% CI: 10%‐38%) and D42 (prevalence 6%; 95% CI: 0%‐14%) in the alfalfa group (*P* = .05) while it did not evolve (prevalence 25% at D0; 95% CI: 12%‐38%; prevalence 22% at D42; 95% CI: 9%‐36%) in control group (*P* = .80). This proportion was not significantly different between groups at D42 (*P* = .09). The antrum was the most affected region by hemorrhage at D0 (prevalence 5%, 95% CI: 0%‐10%). At D42 hemorrhage were predominantly observed in the glandular fundus (prevalence 11%, 95% CI: 4%‐19%). The prevalence of hemorrhage in the glandular fundus evolved from 3% at D0 (95% CI: 0%‐7%) to 14% at D42 (95% CI: 3%‐25%) in the control group (*P* = .10), and from 0% at D0 to 6% at D42 (95% CI: 0%‐14%) in the alfalfa group (*P* = .22). Mucosa in relief was rarely observed (9 of 205 observations) throughout the trial. The proportion of mucosa in relief observed at the pylorus increased between D0 (0 of 37 observations) and D42 in alfalfa group (*P* = .05) to reach a prevalence of 12% (95% CI: 1%‐23%) in this group at the end of the trial. A fibrinous area was only observed in 1 horse at D0 and D42.

### 3.3 Relationship between GGD and biochemical composition of the ration at D0

At D0, no significant relationship was observed between energy intake or biochemical composition of the total ration fed before the trial and GGD. However, when focusing on concentrates fed to horses, daily intake of simple sugars from concentrates was significantly (*P* = .01) higher for horses in the nonulcerated group (77.5 g/kg BW; 95% CI: 71.1‐84.0) compared with the ulcerated group (59.1 g/kg BW; 95% CI: 48.0‐70.3). The intake of other biochemical components did not differ significantly between groups (Table 3).

**TABLE 3:** Average dietary intake from concentrates before D0 (means ± SD) and LS‐means comparison between the nonulcerated (glandular scores 0‐1) and ulcerated (glandular scores ≥2) groups at D0 for the intensively exercised trotters.

	Nonulcerated group at D0 (N = 64)	Ulcerated group at D0 (N = 13)	*P*‐value
Dry matter (g/100 kg BW)	1395 ± 423	1198 ± 312	.07
Digestible energy (MJ/100 kg BW)	19.6 ± 6.1	17.0 ± 4.3	.07
Crude proteins (g/100 kg BW)	182 ± 61	175 ± 38	.55
Starch (g/100 kg BW)	459 ± 156	435 ± 108	.24
Simple sugars (g/100 kg BW)	78 ± 26	59 ± 21	.01
NDF (g/100 kg BW)	378 ± 95	327 ± 93	.40
ADF (g/100 kg BW)	218 ± 75	148 ± 56	.20
Calcium (g/100 kg BW)	14.9 ± 6.1	12.5 ± 5.1	.43
Phosphorus (g/100 kg BW)	8.1 ± 2.5	7.6 ± 2.2	.22

Abbreviations: ADF, acid detergent fibers; BW, body weight; NDF, neutral detergent fibers.

### 3.4 Effect of diet on GGD evolution after 21 and 42 days

**FIGURE 3:**
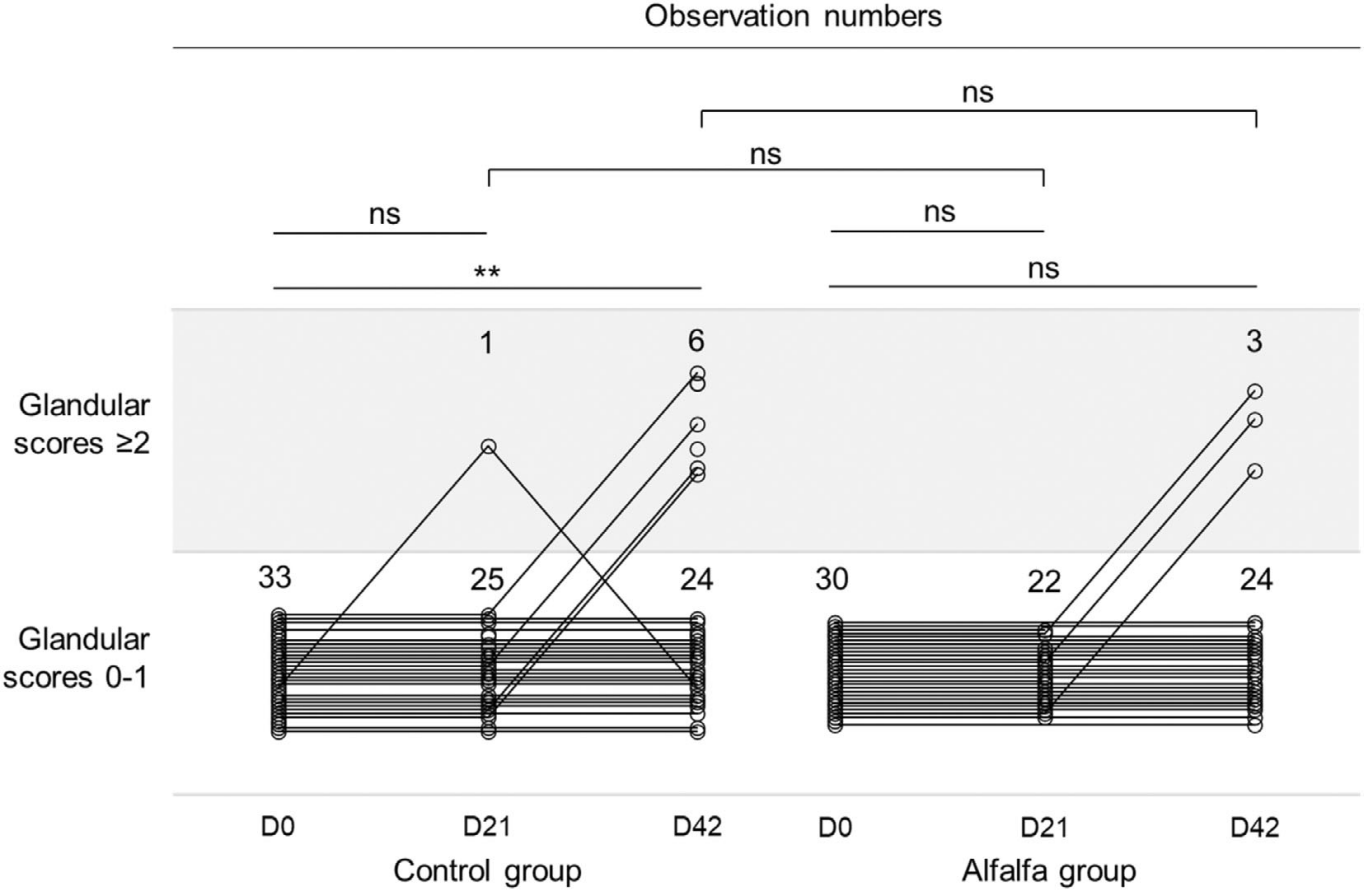
Evolution of glandular scores after 21 and 42 days of control diet (hay ad libitum +5.7 ± 1.6 kg DM concentrates) or alfalfa diet (hay ad libitum +3.0 ± 0.8 kg DM concentrates +2.8 ± 0.6 kg DM alfalfa pellets) among the intensively exercised trotters that were in the nonulcerated group (glandular scores 0‐1) when entering the trial. Horizontal lines indicate test cut‐off. * *P* < .05; ** *P* < .01; ns, nonsignificant.

Among the horses that were categorized in the nonulcerated group at D0, the proportion of ulcerated horses did not differ between the control and the alfalfa groups at D21 (prevalence 4% in control group; 95% CI: 0%‐12%; prevalence 0% in alfalfa group; *P* = 1.00) and D42 (prevalence 20% in control group; 95% CI: 6%‐34%; prevalence 11% in alfalfa group; 95% CI: 0%‐23%; *P* = .4; Figure 3). In the control group, the proportion of ulcerated horses was significantly higher at D42 than at D0 (*P* = .01), while this proportion did not differ between D42 and D0 in the alfalfa group (*P* = .10).

**FIGURE 4:**
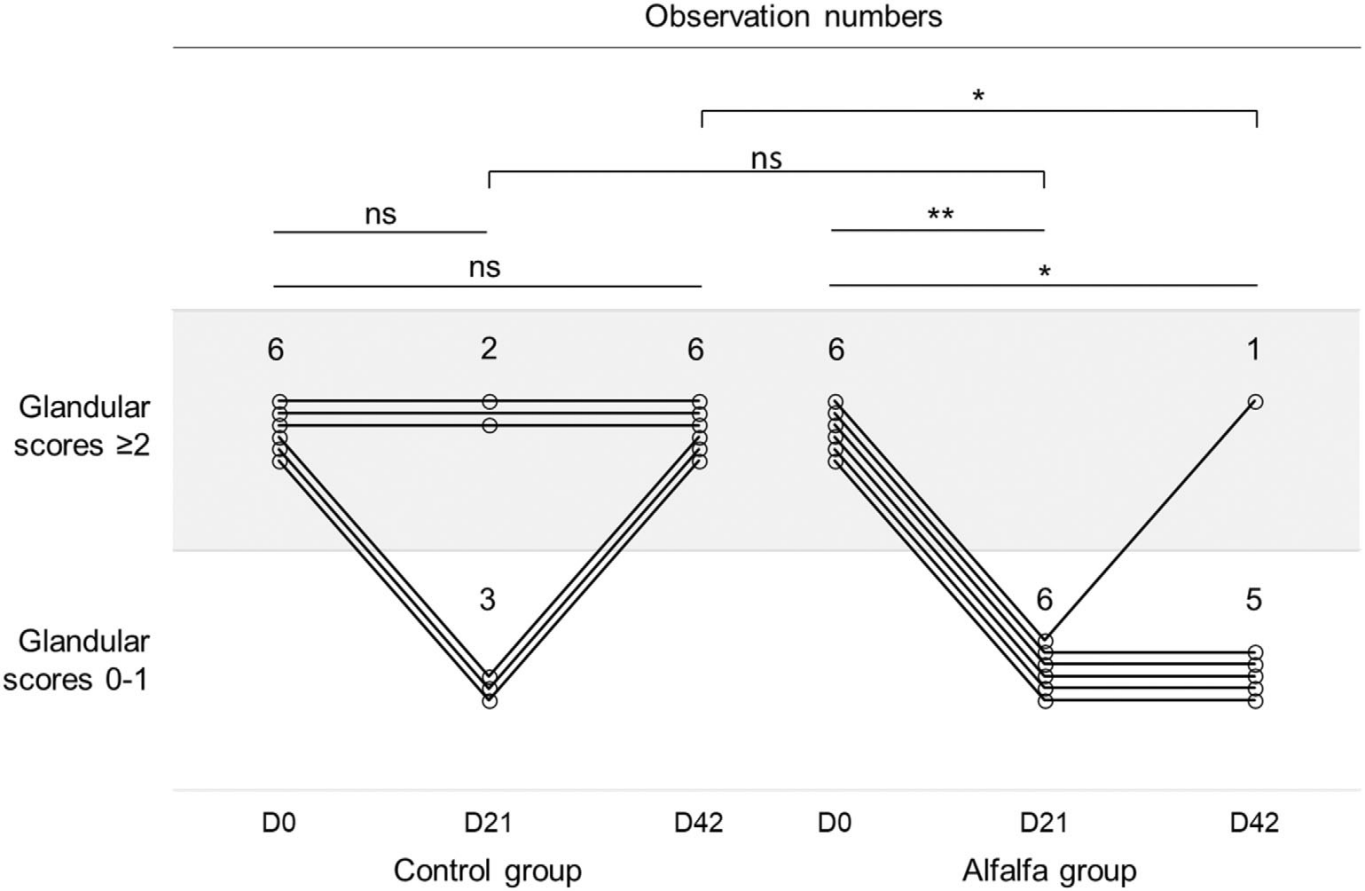
Evolution of glandular scores after 21 and 42 days of control diet (hay ad libitum +5.7 ± 1.6 kg DM concentrates) or alfalfa diet (hay ad libitum +3.0 ± 0.8 kg DM concentrates +2.8 ± 0.6 kg DM alfalfa pellets) among the intensively exercised trotters that were in the ulcerated group (glandular scores 2‐4) when entering the trial. Horizontal lines indicate test cut‐off. * *P* < .05; ** *P* < .01; ns, nonsignificant.

Thirteen horses entered the ulcerated group at D0. One was excluded because its consumption was lower than half of the amount of alfalfa distributed daily. Among the 12 remaining horses, no difference between the 2 groups was observed at D21 (*P* = .18). At D42, a lower number of horses with glandular scores ≥2 was observed in the alfalfa group (1 of 6) compared with the control group (6 of 6; *P* = .02; Figure 4). Clinical success was 47.7 times more likely in horses fed alfalfa than in the control group (95% CI: 1.6‐1422.8).

## 4 DISCUSSION

Among the different scoring systems of the equine glandular mucosa, we decided to use a system based on a 0 to 4 notation.^18^ To limit errors and biases related to endoscopic scoring, the glandular mucosa in the body of the stomach was observed before that of the pylorus.^19^ In addition, only horses where the glandular mucosa (including antrum and pylorus) was fully visible were included, and the same experienced veterinary practitioner scored all the horses. With these criteria, the prevalence of GGD scores 2 to 4 that we observed in the cohort of intensively exercised trotters at the beginning of the trial was lower (17%; 95% CI: 9%‐25%) than that reported in samples of sport horses (31%‐51%),^3^, ^4^ and within the lower ranges measured in racehorses at training (17%‐65%).^1^, ^2^, ^20^ The antrum was the most frequently injured region of the glandular mucosa, followed by the pylorus and the fundus. This is consistent with previous observations of horses in training, where all individuals with GGD scores ≥2 had lesions in the pylorus or antrum.^3^ This also concords with results on horses that were examined after exhibiting clinical signs, where 8% of horses had GGD scores ≥2 on the glandular body and 59% on the antrum or pylorus.^21^ During the gastroscopies, we also observed macroscopically that the antral and pyloric regions presented the most frequent congestion. This might reflect mucosa inflammation, which is common in horses.^22^ In this field trial, it was impossible to perform gastric biopsies that would have allowed histological observations and further explanations.

Our data highlight that a higher daily intake of simple sugars supplied by the dietary concentrates was significantly associated with a less severe score of glandular ulcers at D0 in a group of intensively exercised trotters. The other biochemical variables of feed, including the amount of starch, did not have a significant association. Glandular gastric disease might be influenced by the biochemical composition of the ration,^4^, ^23^ but the potential protective effect of simple sugars has not been noted. This might be related to the protective role of certain *Lactobacillus* strains, that is the predominant bacterial genus in equine stomach luminal content,^24^, ^25^ and which is also detected in glandular mucosal biopsies.^26^, ^27^ These bacteria can adhere to the equine gastric mucosa, form biofilms,^28^ and counteract the development of pathogenic or proinflammatory bacteria.^29^ In a rodent model with glandular ulcers induced by the application of acetic acid, boosting the development of Lactobacillaceae by providing lactulose to rats favored the colonization of ulcerated areas in the glandular mucosa by lactobacilli and accelerated healing.^30^ In addition, *Lactobacillus* sp. given to rats as a single probiotic strain (*Lactobacillus gasseri OLL2716*, *Lactobacillus acidophilus*, or *Lacticaseibacillus rhamnosus GG*) or in the form of probiotic mixtures promotes glandular ulcer healing.^31^

We observed a positive relationship between the feeding of alfalfa pellets for 42 days and the decreased number of horses with GGD. In addition to the glandular scores that were lower when horses were fed alfalfa, the proportion of horses that presented congestive mucosa in the antrum also decreased from D0 to D42. Substituting half of the high‐starch pellets with alfalfa pellets reduced the amount of starch (4.4 ± 1.5 vs 2.4 ± 0.8 g/kg BW/day) available for specialized starch‐utilizing bacteria living in the gastric luminal content. This probably altered the production of fermentation end products as evidenced by lactic acid and other SCFAs concentrations that increase in the gastric luminal content by 30‐ and 4‐fold, respectively, compared with preprandial values after feeding a 2.6 kg meal containing 23% starch.^32^ Though lactic acid might not cause damage to tissue sodium transport or barrier function,^33^ its high concentration contributes to the acidification of the stomach environment. Below pH 4.8, the other SCFAs are mostly present in their undissociated form (R‐COOH) and carry harmful effects demonstrated in vitro on the squamous mucosa of the equine stomach.^34^, ^35^ However, only the effects of butyric acid have been studied on the glandular mucosa of the equine stomach and in vitro induce a decrease in tissue conductance and acute swelling with superficial epithelial detachment.^8^ It is suggested that glandular lesions might not develop because of acidification alone but that butyric acid could be the causative agent for functional and morphological mucosal damage.^8^ Similar glandular alterations with deep penetrating ulcers are observed in vivo in other monogastric species when acetic acid solutions are injected into the gastric glandular submucosa or locally applied to the serosa.^7^ Several SCFAs could therefore be harmful to the glandular mucosa, particularly the acetic acid produced in high concentration when horses are fed with rapidly fermentable carbohydrates.^12^, ^32^, ^36^ However, the mechanisms by which this might occur remain to be clarified.

Horses fed high‐starch pellets (42% starch) have slower gastric emptying rates than horses fed the same quantity of high‐fiber pellets (22% starch).^37^ In our study, reducing the amount of starch provided by concentrates also possibly reduced the retention time in the stomach. As the increase in fermentation end products is exponential after a meal,^32^ reducing gastric retention time and the amount of fermentable substrates because of low‐starch, high‐fiber feedstuff might reduce the risks of ulceration.

Because of its high calcium concentration, alfalfa carries a high buffering capacity compared with other common feedstuffs. In vitro, 2.6 to 4.3 times more hydrochloric acid is needed to decrease the pH from 7.0 to 4.0 when dehydrated alfalfa is added to distilled water compared with corn, oats, and barley.^11^ This acid buffering capacity of alfalfa can also contribute to maintaining a higher pH despite the acidic secretions and strong fermentations in the gastric environment, as illustrated in vitro and in vivo.^12^, ^13^

The prevalence of GGD scores ≥2 at D0 was lower than we expected. Therefore, the size of the control and alfalfa groups with ulcerated horses was small (N = 6 per group). Despite these limited numbers, a significant effect of the diet was observed. We did not follow horses with grade 4 GGD at D0, which could have been more challenging. A curative effect was already suggested in a study on 9 nonexercised horses.^15^ However, in that study, there was no control group and no differentiation in the effects on squamous or glandular mucosa. Finally, as observed in weanling horses, the distribution of alfalfa in the form of pellets did not seem deleterious on the pylorus.^16^ Pelleting alfalfa reduces particle size, which likely has a positive effect in limiting highly lignified stems that might be abrasive for the mucosa.

## CONFLICT OF INTEREST DECLARATION

Authors declare no conflict of interest.

## OFF‐LABEL ANTIMICROBIAL DECLARATION

Authors declare no off‐label use of antimicrobials.

## INSTITUTIONAL ANIMAL CARE AND USE COMMITTEE (IACUC) OR OTHER APPROVAL DECLARATION

The protocol was approved by the local ethics committee for animal experimentation (Comité d'éthique de l'expérimentation animale grand campus Dijon) under the number 2019589602.

## HUMAN ETHICS APPROVAL DECLARATION

Authors declare human ethics approval was not needed for this study.
